# PROTOCOL: Financial coaching for enhancing household finances and health/well‐being: A systematic review and meta‐analysis

**DOI:** 10.1002/cl2.70012

**Published:** 2024-12-11

**Authors:** Julie Birkenmaier, Brandy R. Maynard, Hannah Shanks, Elizabeth Greer

**Affiliations:** ^1^ Saint Louis University School of Social Work St. Louis Missouri USA

**Keywords:** financial coaching, financial outcomes, financial stress, health outcomes, well‐being

## Abstract

This is the protocol for a Campbell systematic review. The objectives are as follows. The primary objective of this review is to answer the following research questions: (1) What is the extent of financial coaching intervention research? (2) What are the effects on financial outcomes of financial coaching embedded within community settings? (3) What are the effects on financial outcomes of financial coaching embedded within healthcare settings? (4) What are the effects on health/well‐being‐related outcomes of financial coaching embedded within community settings? (5) What are the effects on health/well‐being‐related outcomes of financial coaching embedded within healthcare settings? (6) What study or intervention characteristics are associated with variation in the effects of financial coaching (i.e., design (RCT and QED), publication status (published or unpublished), dosage and duration of financial coaching intervention (continuous variable), age, financial coaching elements, and setting of intervention (healthcare or non‐healthcare)?

## 1 BACKGROUND

### 1.1 U.S. household financial stress

Household financial stress, or difficulty meeting basic financial commitments, is a widespread and significant problem in the United States, affecting a variety of populations (Board of Governors of the Federal Reserve System, 2024). About 38 million people live in households below the U.S. poverty line (U.S. Census Bureau, 2022), and many more live just above that line. About 50 million Americans have households with incomes below 125% of the federal poverty line, including more than 15 million children (Legal Services Corporation, 2024). Beyond those in and just above poverty, financial stress also affects households with negative income variability, those experiencing and anticipating sudden large expenses, and those with health issues that cause unmanageable debt, among others (Board of Governors of the Federal Reserve System, 2024).

Inadequate income is a special concern for minoritized populations, as poverty disproportionately impacts racial and ethnic minorities (Board of Governors of the Federal Reserve System, 2024). Even those households that are able to meet daily and monthly expenses experience financial stress when an unexpected expense occurs. Most U.S. households have less short‐term savings than the amount needed to cover expenses for 3 months in the absence of income, as recommended by financial planners (FINRA Investor Education Foundation, 2019). In 2023, only 63% of adults could cover a hypothetical expense of $400 using cash, savings or a credit card (and they are able to pay off the $400 charge at the next statement) (Board of Governors of the Federal Reserve System, 2024). Households with financial need, but without savings, often take on debt. Fully 28% of the population has taken on unmanageable debt that has resulted in debt collections documented on their credit report (Carther et al., 2022). The lack of savings is also critical for older adults; only about one‐third of non‐retirees think that their retirement savings is on track (Board of Governors of the Federal Reserve System, 2024).

### 1.2 Health/well‐being effects of poverty and financial strain

The long‐term health/well‐being effects of poverty and financial stress and financial strain, or the physical results of financial stress, are well‐documented (Shippee et al., 2012). The health problems associated with inadequate income are one of the social determinants of health, which refers to a broad range of social and environmental conditions that affect health/well‐being (Francis et al., 2018; U.S. Department of Health and Human Services, n.d.). Beyond financial implications, living with inadequate income and financial stress has major effects on people's physical health because poverty affects a person's access to safe housing, healthy foods, employment and educational opportunities, healthcare services, clean air and water, and a safe neighborhood environment. Inadequate income affects both adults and children: growing up without access to these resources negatively affects children's health/well‐being (Fuji et al., 2024; U.S. Department of Health and Human Services, n.d.). The effect of finances on health has been demonstrated at the policy level as well: Bradley et al. (2016) demonstrated that states with more social services that affect income and finances had significantly better health outcomes.

Financial stress and resulting financial strain negatively affects health/well‐being through several pathways. First, chronic stress leads to the acceleration of normal aging through shortening of telomeres and increases in allostatic load. These processes, called “weathering,” lead to earlier onset of unfavorable physical health conditions (Forde et al., 2019). Second, financial stress disrupts medical care due to foregone care as a result of families neglecting their health because they cannot afford to seek preventative or early care, or experience missed clinic visits due to cost or other poverty‐related access challenges (Samuels et al., 2015). Financial stress can force families to focus on basic needs that may be more urgent than preventive medical care visits, which often results in worsening symptoms and thus requiring more expensive or prolonged care (Schickedanz et al., 2023). Children in low‐income households whose parents are under financial stress may suffer harm to their cognitive and social/emotional development due to harsh, inconsistent and detached parenting (National Academies of Sciences, Engineering, and Medicine, 2019). Additionally, some health conditions are associated with financial stress, such as tobacco use as a stress coping mechanism (Centers for Disease Control and Prevention [CDC], 2011).

### 1.3 Financial exploitation and financial decision making

Financial strain is also a result of financial exploitation, and a growing public policy and public health concern. Financial exploitation is defined as “the wrongful taking, withholding, appropriating or use of a vulnerable adult's money, real property, or personal property” (U.S. Department of Justice, n.d., HRS 346‐222, 2022). Estimates vary on the number of older adults who have been victimized, but it is the second most common type of crime for older adults (23%) (U.S. Department of Justice, Office for Victims of Crime, 2023). Consumers aged 60 and older reported losses of $1.6 million in 2022 to scams, with investment scams representing the type of scam with the most losses (Federal Trade Commission, 2024). Other estimates are much higher – the National Council on Aging (2024) estimates that financial exploitation causes $28.3 billion in losses each year (National Council on Aging, 2024).

Financial institutions reported a fourfold increase in the reporting of suspicious banking activities for consumers over age 60 between 2014 and 2017 (Consumer Financial Protection Bureau [CFPB], 2019). Older adults often experience difficulty cleaning up the aftermath of exploitation by making reports to the appropriate agencies and put protections in place to prevent future exploitation (Wood & Lichtenberg, 2017). Financial exploitation of older adults is reported to affect their finances and is associated with poorer health, including poorer memory, executive functioning, social support and stress levels (Hall et al., 2022).

### 1.4 The intervention

The earliest comprehensive descriptions and studies of financial coaching were published after 2007 (Collins et al., 2007). These early descriptions and studies focused on the financial coaching in the asset‐building field, as well as other financial work with low‐wage workers in efforts to improve their financial behaviors (Collins et al., 2007; Managan, 2010). While there is still no evidence‐based industry standard for financial coaching (Collins & Olive, 2016), most financial coaching programs generally follow the model set forth by Collins and O'Rourke (2012). Financial coaching has grown in recent years as a strengths‐based, future‐oriented, collaborative approach that uses client goals setting and behavioral practice to assist low‐income families to navigate financial issues for better financial and/or health outcomes. Rather than focused on a specific event or problem, coaching is focused on helping low‐income families in achieving their potential for financial well‐being (Center for Financial Security, 2015; Hall et al., 2022; Modestino et al., 2019; Schickedanz et al., 2023).

Financial coaching is a structured, collaborative process to help participants bridge the gap between intention and knowledge, and create lasting behavior change. In the coaching model, participants set goals meaningful to them, commit to taking certain actions within specific timeframes, and agree to be held accountable by the coach for taking the actions (Collins & O'Rourke, 2012; Collins et al., 2021; Managan, 2010). Key elements of coaching include a focus on long‐term outcomes, and building skills based on the client's unique needs and goals (Collins et al., 2007). Financial coaching is typically used with clients in relatively stable situations and over a longer period of time (median of six times over weeks and months at intervals of the client's choosing) than financial education or counseling (Consumer Financial Protection Bureau [CFPB], 2016; Lienhardt, 2016). Financial education is an optional component within financial coaching that can be added to the practice model, but it cannot replace the key elements of coaching ‐ goal setting, action planning, and follow‐up (Collins et al., 2013).

Financial coaches focus on action planning and reinforcing self‐control by increasing financial confidence, knowledge, and behaviors while reducing financial stress (Collins et al., 2021). Financial coaching stands in contrast to financial education or counseling that provide prescriptive information and advice (Silva et al., 2022). It is solution‐ and result‐focused, and strengths‐based to enhance personal financial management. Key to the model is client accountability for planned actions, and coach follow‐up with the clients (Collins et al., 2021).

### 1.5 Populations, settings, and intervention modes

Financial coaching is implemented in many different settings, with various populations, and individually, in groups, and in a mix of individual and group formats. The early literature and studies of financial coaching focused on low‐wage workers and low‐income households with one‐on‐one service delivery, and in community‐based settings (Collins et al., 2013; Managan, 2010; Packard et al., 2015; Roder, 2016; Theodos et al., 2015; White et al., 2015, 2018). Youth in government‐sponsored workforce development programs were also the subject of early attention (Loke et al., 2016; Modestino et al., 2019), along with lower income homeowners (Moulton et al., 2015).

Later studies continued the focus on lower‐income populations (Fuji et al., 2024), as well as different populations. For example, Hall and Lichtenberg (2022, 2024) focused on older adults who had been victimized by financial exploitation. Healthcare settings serving clients with financial stress have also created Medical‐Financial Partnerships to provide financial coaching within healthcare settings (Bell et al., 2020; Schickedanz et al., 2023). Researchers are also experimenting with financial coaching in specialty healthcare, such as smoking cessation programs (Rogers et al., 2022). Coaching has also been implemented with other languages beyond English, including Spanish (Consumer Financial Protection Bureau [CFPB], 2016; Schickedanz et al., 2023). Financial coaching has predominantly been delivered individually, but a few studies have highlighted small group‐based financial coaching (Baker & O'Rourke, 2013; Loke et al., 2016; Silva et al., 2022).

### 1.6 How the intervention might work

While other interventions to improve financial well‐being and reduce financial stress take different approaches (e.g., Birkenmaier et al., 2022), financial coaching is directly focused on changing consumer behavior toward financial outcomes that will reduce financial stress and improve financial well‐being (Collins & Olive, 2016; Theodos et al., 2015).

The first step in individual financial coaching is to meet with the client(s). Although there are specific tasks to be completed, a major focus is to build supportive, trusting, nonjudgmental relationships to support client capabilities and strengths to reduce barriers to continued engagement (Schickedanz et al., 2023). Therefore, the coach may take time to get to know the client on a personal level throughout the interaction. Participants often complete an intake form or survey before or just after program enrollment to gather vital sociodemographic information and sometimes assess eligibility for coaching (Rogers et al., 2022). This intake or assessment form may assess income, including public benefits eligibility or receipt, savings, credit, debt, taxes, and whether the household has and uses a budget (Schickedanz et al., 2023).

Either in the same or subsequent meetings, the coach and client create, discuss, and possibly refine the client's financial goal(s) to be actionable and achievable. The coach helps the participant to define and articulate their competencies, resources, and desires. Next, the coach helps the client break down the goals into several smaller steps, with the participant committing to complete each step by a particular date. The coach and participant may meet in person, telephone, or via video conferencing only once, with follow‐up by the same or different methods over a period of time chosen by the participant or the program. The coach may also contact the client in‐between sessions to ask about their progress in accomplishing tasks (Schickedanz et al., 2023). In the process of helping the client to achieve their goals, coaches may provide the necessary financial education and assist in improving budgeting, savings, debt and credit, eligibility screening for and assistance with applying for public benefits, financial planning – whatever is needed to achieve the client‐determined financial goal(s) (Collins et al., 2021). There is no standardized number of sessions, length of intervention time, or interval times between contacts. In individual studies, financial coaching has been shown to increase financial knowledge and confidence, reduce financial stress, increase income and savings, reduce debt, and improve credit (Collins et al., 2021; Silva et al., 2022).

Group financial coaching can involve one or two coaches working together (Baker & O'Rourke, 2013). For this intervention, the beginning phase is similar to other modes of group work, in that the group can determine the rules, create a group name, and have some socialization time for members to get to know each other and build group cohesion (Baker & O'Rourke, 2013). During each session, group financial coaching can involve the use of icebreaker activities to gain trust among the members, check‐in on progress toward goals and discussion of the past week's efforts toward the actionable steps needed toward goals, financial education, activities to reinforce goal selection and enhance motivation, and action planning for the next week (Baker & O'Rourke, 2013). In some models, coaches select some financial education content (e.g., financial values and attitudes, budgeting, credit), and participants select some (e.g., debt reduction, retirement savings, investments) (Baker & O'Rourke, 2013).

### 1.7 Financial coaches

Financial coaches are full‐ and part‐time employees of agencies, trained agency volunteers, and trained peers (Baker & O'Rourke, 2013; Loke et al., 2016). They are typically trained for the role, and may follow a specific curriculum (Theodos et al., 2015). For both individual and group models, the coaches role is as a facilitator to keep the discussion on topic, provide information and education, and provide resources during and in between sessions (Baker & O'Rourke, 2013). They provide ideas and possible solutions, rather than directing the clients toward specific actions. They set a positive tone that is focused on strengths and solutions. The use of trained peer‐coaches as group facilitators is also highlighted in the literature (Loke et al., 2016).

#### 1.7.1 Theory of change

Financial coaching draws from coaching psychology theories (e.g., goal orientation and cognitive coaching) and adult learning theory about self‐directed learning (Collins & Olive, 2016; Collins et al., 2013). Collectively, these theories proport that clients have the capacity to set goals, build self‐regulation and self‐determination, and correct thought, belief, and attitude distortions that affect financial decision‐making (Collins & Olive, 2016). In sum, clients have the capacity to build knowledge and skills to solve their own problems through self‐directed learning (Whitmore & Gaskell, 2024).

To implement these ideas, Collins and Olive (2016) suggest the A4 model. For the first A, alliance, a collaborative relationship is built through discussion of expectations, accountability, and the coaching process. Alliance is also an ongoing process throughout the engagement. The second A is agenda building through the discussion of potential achievable goals, and the segmentation of goals into steps. The third A, awareness, is achieved through the client's expectations, values, barriers, and supports related to their goal(s). Rather than moving quickly to action steps, the coach facilitates a discussion to increase the client's self‐awareness through active listening and validation to explore satisfaction and ambivalence. Action is the fourth A. In this stage, exploring if/then possibilities for action steps to anticipate opportunities and potential roadblocks. Goal setting focuses attention and enhances performance (Collins & Olive, 2016).

Although models vary among financial coaching programs, empowerment of the client is a common theme. Because the coach acts as an equal, participants define their own vision of success, and are in charge of all decisions. Through the experience of continual decision‐making in the presence of an active listener who asks critical questions and positions themselves as a resource, rather than an authority figure, clients experience increased confidence in their ability to make better financial decisions (Managan, 2010; Modestino et al., 2019). Being held accountable to a third party enhances self‐control and helps keep intended behaviors in the forefront of participants' minds, and motivates sustained behavior change (Collins et al., 2013). Motivation and action emerge from the client's increased self‐awareness and self‐responsibility (Whitmore & Gaskell, 2024). In these ways, coaching helps people overcome procrastination and lapses in focus and self‐control toward the goal of behavioral change (Collins et al., 2013).

### 1.8 Health/well‐being‐related theory of change

The connections between financial stress and health/well‐being include the ability to “expand one's bandwidth,” or ability to focus on a particular area. Increased reserves of self‐control are freed up when financial stress is lowered, which allows people to successfully adopt healthy behaviors, including improved diet, increased exercise, and decreased smoking and alcohol use. These changes can lead to improvements in objective health outcomes, including weight, blood pressure, and cholesterol. Decreased stress and improvements in perceived hope and quality of life will decrease the release of stress hormones, including cortisol, norepinephrine, and epinephrine, which also contributes to improvements in health outcomes and overall well‐being (White et al., 2019).

### 1.9 Financial exploitation

Individual study results suggest that financial coaching may impact vulnerability to financial exploitation (Hall & Lichtenberg, 2022, 2024; Wood & Lichtenberg, 2017). Older adults are often the target of financial exploitation for several reasons. They often have retirement savings accumulated over their lifetimes, and make important financial and health‐related decisions, which makes them attractive targets for exploitation. Financial exploitation includes cognitive, financial, emotional, and contextual factors. Contextual factors include the context of the financial decisions and individual differences such as susceptibility to undue influence or excessive persuasion. These factors influence the intellectual factors associated with decisional abilities. Cognitive abilities underlie sound financial management and declines in these abilities compromise good financial decision‐making, which results in susceptibility to financial exploitation (Consumer Financial Protection Bureau [CFPB], 2019; Hall & Lichtenberg, 2022, 2024). The increase in knowledge and self‐efficacy regarding exploitation resulting from financial coaching may result in hoped‐for outcomes of decreased vulnerability.

### 1.10 Why it is important to do the review

Exploring the effectiveness of financial coaching across many settings on financial and health/well‐being outcomes is vitally important toward gaining the evidence needed to make decisions about interventions that effectively change consumer financial behavior and financial stress. Currently, the main practice and policy focus regarding consumer financial behavior is on financial education. Federal agencies, banks, educational institutions, workplaces, community‐settings, and others collectively spend $670 billion dollars on financial education as a primary method of influencing consumer financial behavior in schools, workplaces, job training, and other community settings (Consumer Financial Protection Bureau [CFPB], 2012). Financial literacy is a required element for youth workforce development programs under the 2014 Workforce Innovation and Opportunity Act (WIOPA) (Bird et al., 2014). Currently, more than two‐thirds of all states now require financial education in high schools (Council on Economic Education, 2024). Despite the emphasis and resources spent on financial education, research has shown mixed results. While one study found meaningful treatment size (Kaiser et al., 2022), other systematic review studies have concluded that the effects of such programs are very small (Fernandes et al., 2014; Kaiser & Menkhoff, 2020) or affect only some outcomes (e.g., savings behavior) (Miller et al., 2015) or have highly heterogeneous impacts (Kaiser & Menkhoff, 2016).

At the same time, financial coaching is becoming increasingly common in related human service practice (Loomis, 2018), and the number of individual studies in a widening area of practice is growing. At the last census of the financial coaching field in 2015, there were over 40 coaching organizations, with over 2000 coaches in the United States, and those numbers are only likely to be larger in 2024 (Lienhardt, 2016).

For‐profit financial coaching companies are doing a strong business and growing (e.g., Abundance University, 2024); however, no systematic review or meta‐analysis was located that examines whether financial coaching leads to improved financial outcomes. One 2015 literature review (Center for Financial Security, 2015) summarized research about the theories underlying financial coaching and the effects of financial coaching on participant behaviors and outcomes. Peeters et al. (2018) conducted a literature review on the contents, form, and effectiveness of group‐based programs for combined financial education and counseling, and included group financial coaching. However, a clear delineation was missing between financial counseling and financial coaching. Therefore, evidence is slim on the effectiveness of financial coaching with various populations, in various settings, and for financial and health/well‐being outcomes. With increasing numbers of interventions, settings, and populations exposed to the intervention, it is important to synthesize the evidence to inform practice and policy.

#### 1.10.1 Relevance to health policy

This review has relevance to health policy as well. First, health policy is increasingly recognizing the social determinants of health, such as finances, as important influences on health (American Academy of Family Physicians, 2021; American Academy of Pediatrics, 2023; World Health Organization, 2023). Addressing financial goals and needs of patients, which could lead to improved health outcomes, may impact policy through reduced healthcare costs that would otherwise result from forgone preventative healthcare, such as vaccinations and health screenings. Health policy, especially related to primary healthcare settings, could be influenced by study outcomes. Second, there is currently little evidence supporting the idea that financial coaching could magnify the effectiveness of health services by becoming embedded in frequented and trusted settings, such as health clinics (Alexander et al., 2022). Evidence related to the effectiveness of embedding financial services could shape policy related to these services. For example, evidence of the effectiveness of financial coaching within medical financial partnerships and smoking cessation services could encourage policymakers to incorporate them more fully into health systems and create reimbursement avenues for financial services.

## 2 OBJECTIVES

The primary objective of this review is to answer the following research questions:

1. What is the extent of financial coaching intervention research?
2. What are the effects on financial outcomes of financial coaching embedded within community settings?
3. What are the effects on financial outcomes of financial coaching embedded within healthcare settings?
4. What are the effects on health/well‐being‐related outcomes of financial coaching embedded within community settings?
5. What are the effects on health/well‐being‐related outcomes of financial coaching embedded within healthcare settings?
6. What study or intervention characteristics are associated with variation in the effects of financial coaching (i.e., design (randomized controlled trial [RCT] and quasi‐experimental design [QED]), publication status (published or unpublished), dosage and duration of financial coaching intervention (continuous variable), age, financial coaching elements, and setting of intervention (healthcare or non‐healthcare)?

## 3 METHODS

This review will follow the Campbell Author Guidelines and the MECCIR conduct and reporting standards (The Methods Group of the Campbell Collaboration, 2017).

### 3.1 Criteria for considering studies for this review

#### 3.1.1 Types of studies

We will include both RCTs and QEDs to provide a comprehensive analysis of the available evidence. RCTs are often regarded as the gold standard for determining causality because they reduce selection bias through random assignment. However, we recognize that RCTs may not always be feasible with financial coaching interventions. QEDs, which approximate experimental conditions, allow us to include studies conducted in more naturalistic environments or when RCTs are not feasible. By incorporating both RCTs and QEDs, we aim to capture a wider range of evidence and include a broader range of interventions and more diverse contexts and populations and provide a more comprehensive picture of the state of the evidence.

We will include randomized and quasi‐experimental study designs that meet the following criteria:

1. RCTs – studies that use a random assignment of participants to condition, and evaluates the effect by comparing the outcome between the treatment and control groups.
2. QEDs – When the assignment to groups is not random, studies must use a design that compares a group of participants who receive the intervention and another group that does not receive the intervention. The treatment and control groups must be observed during the same period of time (to avoid a time confound) and must demonstrate baseline equivalence on outcomes (except for those where a baseline measure would not be expected or feasible, such as tax refund receipt and amount which are typically not measured before an intervention) and baseline equivalence on the following participant sociodemographic characteristics: race/ethnicity, household income, and age. Satisfying the baseline equivalence standard requires baseline group differences no greater than 0.25 standard deviation on the key covariates. Where differences are between 0.05 and 0.25 standard deviation, study authors must statistically adjust for those baseline covariates in their outcome analyses. For studies that do not meet this criteria, the studies will be included in the review, but not in the meta‐analysis.

The comparison group for both RCTs and QED studies could include those on a waitlist, attention control, or standard care. For studies that involve healthcare settings, comparison or control groups can receive their needed healthcare, but no financial coaching within the healthcare setting. It is possible that comparison/control group members receive financial coaching outside of the setting.

#### 3.1.2 Type of participants

We will include studies with participants from any population (e.g., children and adults of any age).

#### 3.1.3 Settings

All non‐healthcare community settings within and outside of the United States (e.g., government agencies, community centers, service agencies) will be included. We will limit interventions that take place in a healthcare setting to the United States only. We will exclude studies where financial coaching is provided within a healthcare setting outside of the U.S.‐based healthcare setting because of the unique nature of the U.S. healthcare system among other developed countries.

#### 3.1.4 Type of interventions

The eligible intervention will be the provision of financial coaching for individuals that includes client‐led financial goal setting, action planning, and/or monitoring of progress with the aim of improving financial habits/practices and overall financial well‐being (Collins et al., 2013). The goal of the financial coaching must be for personal, rather than business, finances. Because financial coaching is not an accredited activity and no universal standards exist, the term “financial coaching” could be used to refer to an array of approaches. Therefore, to create a common term for this review, this study sets criteria for necessary components of financial coaching to delineate it from financial education, financial capability, financial counseling, and other related terms. While financial coaching can include many discrete or adjunct services (Roder, 2016), only studies that *explicitly discuss* at least two of the three criteria in their financial coaching intervention description (client‐led goal setting, action planning, and progress monitoring by the coach) will be included. Financial coaching could be provided by any group, organization, or agency, whether non‐profit or for‐profit, whose primary role is to provide such services.

##### Excluded studies

Similar interventions that are not eligible are:

1. Interventions that provide financial coaching that are primarily aimed at repayment to a particular merchant (e.g., credit card company) (Ibarra et al., 2017).
2. Interventions that are aimed to assist business owners, rather than individuals.
3. Interventions in healthcare settings that are delivered for the sole purpose of affording healthcare and/or medications. These interventions, such as cash assistance to purchase medications or afford copays, or programs that discount medications or the cost of medical services, are solely focused on delivering the needed healthcare services, and not on improving the patient's overall financial situation and reducing financial stress.

#### 3.1.5 Type of outcome measures

##### Primary outcomes

To be included in this review, studies must measure and report at least one financial outcome as a primary outcome. All financial outcomes will be included. These outcomes can include financial knowledge and attitude, tax filing status, tax refund receipt and amount, debt, savings, credit scores, income, amount recovered from a scam, and financial strain. If a study measures a financial outcome, but does not provide sufficient data to calculate an effect size to be included in a meta‐analysis, the study will still be included in the review.

##### Secondary outcomes

If studies meet all other inclusion criteria, we will extract data related to health/well‐being outcomes if reported. All reported health/well‐being outcomes and perceptions about them will be included. These outcomes can include missed visits, immunization schedule adherence and other health/well‐being indicators, including, but not be limited to the following:

1. Neurocognitive outcomes (Hall et al., 2022).
2. Physical health outcomes (Hall & Lichtenberg, 2022, 2024).
3. Emotional health/mental health outcomes (e.g., anxiety, depression, stress) (Hall & Lichtenberg, 2022, 2024).
4. Functional ability measures (e.g., activities of daily living) (Hall et al., 2022).
5. Social support (Hall & Lichtenberg, 2024) (MSOSS).
6. Outcome related to healthcare usage (e.g., absenteeism, physician visits, emergency room visits, hospitalization days) (Fuji et al., 2024; White et al., 2015, 2023).
7. Other outcomes related to health (e.g., tobacco use, weight loss) (White et al., 2023).
8. Adverse or unintended outcomes reported in primary studies.

We are defining these outcomes very broadly and inclusively since there are a wide range of outcomes that are being measured in the studies we have thus far identified in our initial search to test search terms and gauge the number of expected studies. We want to be able to capture all outcomes related to finances as well as health/well‐being outcomes that are being measured.

#### 3.1.6 Duration of follow‐up

We will include outcomes measured at post‐intervention and all follow‐up time points.

#### 3.1.7 Other inclusion criteria

We will include studies that are conducted any time (no time exclusion). We anticipate studies will be dated after 2007, as, to our knowledge, studies on this intervention started to be produced in 2007. However, we will not exclude studies published earlier.

We will include studies irrespective of whether they are published in journals/books or available as working papers, reports, or other.

We will include studies published in English or any other language, conditional on our ability to have the study translated into English.

### 3.2 Search methods for identification of studies

We will attempt to identify and retrieve both published and unpublished studies through a comprehensive search that includes multiple electronic databases, gray literature sources, reference lists of reviews and relevant studies and inquiry of experts, trial registries, and relevant websites (Kugley et al., 2017). The search strategy and results of the search will be documented in sufficient detail to produce a flow chart.

#### 3.2.1 Electronic searches

We will search the following electronic databases (Platform): ABI/INFORM (ProQuest); Academic Search Complete (EBSCOhost), Business Source Premier (EBSCOhost); CINAHL Plus with Full Text, ProQuest Dissertations & Theses Global; EconLit (EBSCOhost); PubMed; APA PsychInfo (EBSCOhost); SCOPUS (Elsevier): Web of Science Core Collection (Clarivate) (Arts and Humanities Citation Index, Social Sciences Citation Index, Science Citation Index, Expanded Emerging Sources Citation Index, and Conference Proceedings Citation Index).

##### Search terms

We will use combinations of terms related to the intervention and study design to search the electronic databases. Database‐specific strategies will be explored for each database in consultation with a librarian at Saint Louis University, including the use of index‐subject terms, truncation, and data‐base specific limiters, and thesauri will be consulted to employ more precise search strategies within each database. We will use any available database filters as appropriate to the database and our review criteria. See Appendix S1A for a sample search in the ABI/Inform (ProQuest) database.

Below are examples of the types of terms we anticipate using:

a. *Related to the intervention*:
  “financial coach” OR “financial coaching” OR “financial coaches” AND
b. *Related to outcomes*:
  (“reduce debts” OR “reduce debt” OR “lower debts” OR “lower debt” OR “build credit” OR “increase credit” OR “build assets” OR “generate wealth” OR “grow income” OR saving OR save OR savings OR delinquent OR delinquency OR delinquencies OR “financial hassles” OR “financial hassle” OR “financial exploitation” OR scam* OR “financial strains” OR “financial strain” OR budget*) AND
c. *Report type:*
  (evaluation OR intervention OR treatment OR outcome OR program OR trial OR experiment OR “control group” OR “controlled trial” OR “quasi‐experiment” OR random* OR empirical OR research)

#### 3.2.2 Searching other sources

Trial registries: We will search the following trial registries: Clinicaltrials.gov, Cochrane Central Register of Controlled Trials, and the International Clinical Trials Registry Platform (WHO; https://who.int/ictrp/network/en/). We will also search the Centers for Disease Control and Prevention website.

Website and online sources: Our search for unpublished studies will include the following relevant websites: World Bank, OECD, Global Partnership for Financial Inclusion, Alliance for Financial Inclusion, Center for Financial Security at the University of Wisconsin‐Madison, Annie E. Casey Foundation, and NeighborWorks America.

The reference lists from included studies and related systematic/scoping reviews will be reviewed for potential studies. We will also conduct forward citation searching using Google Scholar to search for studies citing included studies.

Authors of included studies will be contacted in an attempt to obtain unpublished studies, studies in process, and published studies missed in the database search.

### 3.3 Data collection and analysis

#### 3.3.1 Description of methods used in primary research

Studies examining the effects of these interventions primarily use group design studies to compare differences between groups at the end of the intervention and, in some cases, additional follow‐up time points. For example, Schickedanz et al. (2023) used an RCT comparing clinic‐based financial coaching offered to the treatment group to usual care among low‐income parent‐infant dyads attending pediatric preventive care visits. They studied the outcomes of employment, savings, and public benefits enrollment through the 6‐month well‐child visit.

#### 3.3.2 Selection of studies

One reviewer will conduct the initial search in all sources and will save the search results in an electronic format (Endnotes) and remove duplicates. At this stage, the reviewer will upload the results into Rayyan to examine titles and abstracts and will discard results that are obviously ineligible (nonempirical report, book review, editorial, not human subjects, etc.). Three reviewers will then independently conduct title and abstract screening using a screening instrument (see Appendix S1B) with the blind function turned on. A consensus process will be used among the reviewers to determine those reports to be moved to full‐text review. For those that are not obviously ineligible, a reviewer will move all records to the full text review stage and full texts will be uploaded into Rayyan. Two reviewers will then independently screen each of the reports for eligibility using a screening instrument (See Appendix S1B) in Rayyan with the blind function turned on. Once two reviewers screen all full texts, a third reviewer will compare agreement between coders and identify any discrepancies. The review team will meet to review and discuss discrepancies and will resolve all discrepancies through consensus.

#### 3.3.3 Data extraction and management

For all studies that pass the eligibility screening process described earlier, two reviewers will independently code all eligible studies using a structured data extraction form (See Appendix S1C). Multiple reports on individual studies will be collated, with the most comprehensive report being designated as the primary report and other reports of the same study will be designated as secondary reports. The data extraction form will include items related to bibliographic information and source descriptors: methods and procedures; context, intervention characteristics; sample characteristics; and outcome data needed to calculate effect sizes.

Coders will pilot test the code form together using diverse types of studies and will discuss any items that are unclear and ensure mutual understanding of all items. Following pilot testing of the form, two coders will independently code 100% of the included studies. Coders will compare coding and will identify and discuss discrepancies, which will be resolved through consensus. If consensus cannot be reached between the two coders, a third member of the review team will be consulted to resolve the discrepancy. Initial discrepancies will be recorded and inter‐rater reliability will be reported. In addition to comparing codes between coders, we will also compare the magnitude and direction of effects as presented in the review with how data is presented in the primary study to check for agreement.

#### 3.3.4 Assessment of risk of bias in included studies

Two review authors will independently assess the risk of bias in all included studies. The Risk of Bias in Non‐randomized Studies of Interventions (ROBINS‐I) (Sterne et al., 2016; https://methods.cochrane.org/bias/risk-bias-non-randomized-studies-interventions) will be used for studies that use a non‐randomized study design, and the Cochrane Collaboration's Revised risk‐of‐bias tool for randomized trials (RoB 2) will be used for studies that use a randomized control trial design (Higgins et al., 2024; https://methods.cochrane.org/bias/resources/rob-2-revised-cochrane-risk-bias-tool-randomized-trials).

Using the ROBINS‐I to assess the non‐randomized studies, the review authors will assess the risk of bias for each of the following seven domains: due to confounding, in selection of participants into the study, in classification of interventions, due to deviations from intended interventions, due to missing data, in measurement of outcomes, and in selection of the reported result. Following the structure of the ROBINS‐I, each study will be coded as “low,” “moderate,” “serious,” “critical” risk of bias or “no information” on each of the domains, and overall.

For the RoB 2, the review authors will assess the risk of bias for each of the following five domains: arising from the randomization process, due to deviations from intended interventions, due to missing outcome data, in measurement of the outcome, and in selection of the reported result. Each study will be coded as “low,” “some concerns,” or “high” risk of bias on each of the domains, and overall. Following independent coding by the review authors, discrepancies will be resolved through consensus

Risk of bias in each domain will be reported within and across studies in the results section using narrative and graphs. We do not plan to restrict analyses based on the risk of bias in any domain. We plan to present all included studies, provide the evidence and source used for the judgment, and provide a narrative discussion of the risk of bias to include discussion of the potential limitations of the review as well as implications of bias in the interpretation of the results in the Discussion section of the review.

##### Statistical procedures and conventions

We will conduct descriptive analyses on variables of interest from all included studies to provide information regarding:

- Study participants (e.g., subgroups, gender, race/ethnicity, income level, age).
- Setting where studies are situated (e.g., community agency, pediatric health clinics).
- Funding mechanisms.
- Relevant intervention characteristics (e.g., type/duration of financial coaching).
- Risk of bias across RCT and QED studies on each domain.

#### 3.3.5 Measurement of treatment effect and unit of analysis

Following descriptive analysis, we will estimate the effect sizes for each outcome of interest in each included study using the most detailed numerical data reported by the study authors. If data needed to calculate an effect size is not reported, we will contact study authors to request the data needed. For the RCT and QED studies, we will calculate the magnitude of effect using the standardized mean difference effect size with Hedges' *g* correction for continuous outcomes and odds ratios for outcomes presented as dichotomous variables. We anticipate that outcomes within a category will be measured using similar metrics and therefore, effect sizes within each category will only use either Hedges' *g* or odds ratio metrics in the analysis. If, however, outcomes within one of the outcome categories are measured in different metrics, we will convert them to Hedges' *g*. When studies use a clustering design, we will calculate effect sizes using the Practical Meta‐Analysis Effect Size Calculator (Wilson, n.d.), incorporating adjustments for clustered RCTs (Institute of Education Sciences, 2022). All effect sizes will be coded so that a positive effect size is indicative of the treatment group positively outperforming the comparison group on the outcome. We interpret a statistically nonsignificant *p*‐value (e.g., larger than 0.05) as a finding of uncertainty unless confidence intervals are sufficiently narrow to rule out an important magnitude of the effect.

#### 3.3.6 Criteria for determination of independent findings

We plan to extract all relevant effect sizes from the primary studies for financial as well as health/well‐being outcomes. We anticipate some studies may report multiple measures of financial outcomes and health/well‐being outcomes that may be measuring conceptually distinct outcomes (e.g., financial attitudes and knowledge vs. money saved). In those cases, we will categorize and synthesize those outcomes separately (one effect size per category). For studies that report multiple intervention groups, we will extract data from the intervention group that meets eligibility criteria. If more than one intervention group meets inclusion criteria, data from all groups will be included. For studies that report multiple time points of the same outcome, we will synthesize one effect from each study at similar time points (e.g., post‐test, 3‐month, 6‐month, 12‐month). For studies that report multiple measures of similarly conceptual outcomes or multiple control or comparison groups, we will include all effect sizes from each study (see Section 3.3.8 for how we plan to synthesize dependent effect sizes).

#### 3.3.7 Dealing with missing data

If primary studies do not report sufficient data to calculate an effect size, we will contact the study authors and request the data. If we are unable to obtain the data, we will include the study in the review, but it will not be included in the meta‐analysis.

#### 3.3.8 Data synthesis

Following the estimation of individual study‐level effects, we will conduct separate meta‐analyses to pool studies for each outcome construct. A weighted mean effect will be calculated by weighing each study‐level effect size by the inverse of its variance. Random effects statistical models will be used throughout unless a compelling case arises for fixed effect analysis. RCT studies will be pooled separately from QED studies; however, if RCT and QED studies are found to be homogenous, studies will be pooled to allow for greater statistical power. If multiple effect sizes are reported per study (e.g., multiple measures of the same outcome construct or multiple control conditions compared to the same intervention condition), we will conduct a meta‐analysis in R using the *robumeta package* (Fisher et al., 2017) for robust variance estimation (RVE) with small‐sample corrections. RVE accounts for the non‐independent sampling errors arising from the inclusion of multiple effect sizes from the same study. Additionally, if a sufficient number of studies are available, we will implement the correlated and hierarchical effects (CHE) model to account for both correlation and hierarchical structure among effect sizes, following current standards for meta‐analysis modeling. The CHE model provides a more refined estimation by incorporating the dependency structure in a way that aligns with best practices in meta‐analytic methods.

#### 3.3.9 Assessment of heterogeneity

Following the estimation of summary effects, we will conduct a test of homogeneity (*Q* test) to compare the observed variance to what would be expected from sampling error. The *I* ^2^ statistic will also be used to describe the percentage of total variation across studies due to heterogeneity rather than chance. In addition, we will report prediction intervals for each outcome's average effect size to provide a range of potential effects across studies (Borenstein et al., 2017). We will also construct a forest plot displaying study‐level mean effect sizes and 95% confidence intervals for the included studies to provide opportunity for visual analysis of the precision of the estimated effect sizes, detection of studies with extreme effects, and information regarding the heterogeneity of studies.

#### 3.3.10 Assessment of reporting biases

Publication bias will be assessed using funnel plots. If we include studies with multiple effect sizes across conceptually distinct outcome constructs, we will use Egger's sandwich method to test the symmetry of the funnel plot, which accounts for the dependency among effect sizes (Rodgers & Pustejovsky, 2021).

#### 3.3.11 Subgroup analysis

Provided that there are a sufficient number of studies, we will conduct moderator analysis to examine whether characteristics of the study methods and interventions may be associated with effect size. The approach to moderator analysis will be dependent upon the available data. If there are sufficient studies available, we will use meta‐regression for all subgroup analyses, dummy coding the categorical variables and including them with any continuous moderators. Moderating variables of interest include study design (RCT and QED), publication status (published or unpublished), dosage and duration of financial coaching intervention (continuous variable), age, financial coaching elements, and setting of intervention (healthcare or non‐healthcare). We anticipate that most of these studies will be conducted with low income groups, and will include a mix of racial/ethnic groups, thus we do not anticipate being able to conduct moderator analysis related to SES/income or race of participants. but if there are studies that differentiate between different income groups, we plan to conduct moderator or subgroup analyses on household income and race.

#### 3.3.12 Sensitivity analysis

Sensitivity analysis will be conducted to examine the potential effects of outliers.

#### 3.3.13 Treatment of qualitative research

We are not including qualitative research.

#### 3.3.14 Summary of findings and assessment of the certainty of the evidence

We do not plan to include a summary of findings and assessment of the certainty of the evidence.

## CONTRIBUTIONS OF AUTHORS

All authors will contribute to the conduct of the review and preparation of the manuscript. The authors responsible for providing the content and methodological expertise are noted below:

- Content: Julie Birkenmaier has substantial expertise and research/publications in the area of financial capability interventions.
- Systematic review methods: Brandy Maynard has extensive training and experience in conducting systematic reviews.
- Statistical analysis: Brandy Maynard has training and experience in conducting statistical analysis for systematic reviews.
- Information retrieval: Brandy Maynard and Julie Birkenmaier have experience in information retrieval from prior systematic reviews. They have consulted with Rebecca Hyde, an information specialist at Saint Louis University, for the search strategy proposed in this protocol and will continue to consult with her throughout the review process.

## DECLARATION OF INTEREST

We have no potential conflicts of interest. No authors have been involved in the development of related interventions, primary research on financial coaching, or prior published review on this topic. We have not received any funding for this project, nor do we have any plans to apply for funding.

## PRELIMINARY TIMEFRAME

We plan to submit the draft review within 12 months after the protocol has been accepted.

## PLANS FOR UPDATING THE REVIEW

The authors agree to update the review if sufficient new studies and adequate funding to undertake the update become available.

## Supporting information

Supporting information.
