# Supplementary material for: PROTOCOL: Financial coaching for enhancing household finances and health/well‐being: A systematic review and meta‐analysis
Source: Campbell Syst Rev. 2024 Dec 11;20(4):e70012. doi: 10.1002/cl2.70012 (PMC11632200; doi:10.1002/cl2.70012)
Supplement: Supplementary file 1 — Supporting information. [file CL2-20-e70012-s001.docx]

**Appendices**

*Appendix A: Sample search*

*Appendix B: Screening Form*

*Appendix C: Data Extraction Form*

**APPENDIX A**

**Sample Search**

Database: ABI/Inform

Search: Advanced Search

Search Fields: All except full text

Dates: All

Exclusion: Wire feeds

| # | Searches | Results |
| --- | --- | --- |
| 1 | “financial coach” OR “financial coaching” OR “financial coaches” AND (“reduce debts” OR “reduce debt” OR “lower debts” OR “lower debt” OR “build credit” OR “increase credit” OR “build assets” OR “generate wealth” OR “grow income” OR saving OR save OR savings OR delinquent OR delinquency OR delinquencies OR “financial hassles” OR “financial hassle” OR “financial exploitation” OR scam* OR “financial strains” OR “financial strain” OR budget*) AND (evaluation OR intervention OR treatment OR outcome OR program OR trial OR experiment OR “control group” OR “controlled trial” OR “quasi-experiment” OR random* OR empirical OR research) | 188 |

**APPENDIX B**

Screening Form

**Is this study a**

- RCT
- QED with parallel cohort
- None of the above – STOP

**Does this study involve an intervention that explicitly states that it provides financial *coaching*?**

- No – STOP
- Yes
- Unsure

**Does the financial coaching involve at least two of the following elements: 1) client-led financial goal setting; 2) action planning; and 3) follow-up?**

- No - STOP
- Yes
- Unsure

**Does this study measure an outcome related to finances?**

- No – STOP
- Yes
- Unsure

**If the study reports healthcare-related outcomes, is it based on the United States?**

- No – STOP
- Yes
- Unsure

**Is this study eligible for the review?**

- No – Reason _____________
- Yes
- Need more information to make a decision

**APPENDIX C**

Data Extraction Form

**Report type**

1. Journal article
2. Book/book chapter
3. Government report (local, state, federal)
4. Conference proceedings
5. Thesis or dissertation
6. Unpublished report (non-government, technical report) and other
7. Research brief

**Intervention setting**

1. Community (nonprofit, for-profit or government non-healthcare setting)
2. Older adult-specific community setting (nonprofit, for-profit, or government non-healthcare setting)
3. Adult, family or pediatric clinical (outpatient) setting
4. Hospital
5. Ambulatory sites affiliated with a hospital
6. Older adult clinical setting

**Patients/participants are**

1. Children and youth
2. Adults
3. Both

**Financial service provided (check all that apply)**

1. Financial coaching
2. Financial literacy/education
3. Financial counseling
4. Credit/debt counseling/debt reduction services
5. Free tax preparation
6. Matched college savings program
7. Employment services
8. Job training
9. One-on-one case management
10. Public benefits screening
11. Assistance in applying or obtaining public benefits
12. Budgeting assistance
13. Expense reduction
14. Savings promotion
15. FAFSA form assistance

**Financial coaching elements include (check all that apply, need two for eligibility)**

1. Client-led financial goal setting
2. Action planning
3. Follow-up

**Healthcare services provided (check all that apply)**

1. Smoking/tobacco use cessation
2. Weight loss
3. Diabetic services
4. Primary healthcare (vaccinations, etc.)
5. Other (fill in)

**Treatment format**

1. Individual (one-on-one) – customized treatment of some kind
2. Group – same tx for all subjects in tx group(s)
3. Individual and group mix of standard and customized tx
4. Other
5. Not specified

**Length of treatment**

1. Specified:
2. Not specified

**Methods of contact for coaching**

1. In person
2. Telephone
3. Video (e.g., Zoom)
4. Texting
5. All the above
6. Any of the above, depending on client preference
7. Some mix of the above (e.g., in person and texting, etc.)

**Total number of sessions**

1. Specified:
2. Not specified

**Length of each session**

1. Specified:
2. Not specified

**Unit of assignment to conditions**

1. Individual participant
2. Group/cluster: specify:
3. Other
4. Not enough information to determine

**Method of assignment to condition**

1. Random, simple
2. Random, after matching, stratification, blocking, etc.
3. Quasi-random assigned by some naturally occurring process
4. QED with parallel cohorts
5. Not specified/not enough information to determine

**What did the control/comparison group receive?**

1. Nothing or wait list
2. Treatment as usual: specify
3. Specified treatment: specify
4. Other

**Who provided the financial coaching?**

1. Nonprofit staff
2. For-profit company staff
3. Government/public staff
4. Researchers
5. Students
6. Other
7. Not specified

**Financial coaches were trained**

1. Specified (describe):
2. Not specified

**Financial coaches followed a curriculum**

1. Specified:
2. Not specified

**Mean age of participants**

Not specified

**Patients are**

1. Primarily non-English speaking
2. Primary English speaking

**Predominant race/ethnicity**

1. African American
2. Asian
3. White, non-Hispanic
4. Hispanic
5. Not specified

**Sex**

1. Female %
2. Not specified

**Income**

1. Low Income
2. Low and moderate income
3. All income levels
4. Not specified

**Results of statistical comparisons of pretest differences on outcomes**

1. No statistical comparisons made
2. No statistically significant differences
3. Statistically significant differences found

**Results of statistical comparisons of pretest differences on demographics (race, ethnicity, income)**

1. No statistical comparisons made
2. No statistically significant differences
3. Statistically significant differences found

**If groups were non-equivalent at baseline, were statistical controls used?**

1. Yes
2. No
3. Were equivalent
4. Not enough information to determine

**If matching was used, how were groups matched?**

1. Matched on pretest measure
2. Matched on demographics
3. Matched on both of the above
4. Propensity Score Matching
5. Other matching technique
6. Not enough information to determine
7. Were not matched

**Role of the evaluator/author/research team or staff in the program**

1. Researcher independent of treatment – research only
2. Researcher not independent of treatment
3. Not specified

**Is this intervention manualized?**

1. Yes
2. No
3. Not specified

**Did the study measure fidelity?**

1. Yes
2. No

**How was fidelity assessed?**

1. Not measured
2. Researcher observations
3. Interviews of participants
4. Surveys of participants
5. Participant logs
6. Administrative records
7. Checklists
8. Other
9. Not specified

**Did the treatment group have high attrition (for RCT/QED > 20%)?**

1. Yes
2. No
3. Not enough information to calculate

**Did the control group have high attrition (for RCT/QED > 20%)?**

1. Yes
2. No
3. Not enough information to calculate

**Timing of measurement of outcomes**

**Financial outcomes**

1. Tx baseline mean
2. Tx baseline SD
3. Tx baseline N
4. Tx post mean
5. Tx post SD
6. Tx post n
7. Control group baseline mean
8. Control group SD
9. Control group n
10. Values for t, F, other

Tx analytic sample size

Control group analytic sample size

**Health/well-being outcomes**

1. Tx baseline mean
2. Tx baseline SD
3. Tx baseline N
4. Tx post mean
5. Tx post SD
6. Tx post n
7. Control group baseline mean
8. Control group SD
9. Control group n
10. Values for t, F, other

Tx analytic sample size

Control group analytic sample size
